# Cellular and Molecular Mechanisms Mediated by recPrP^C^ Involved in the Neuronal Differentiation Process of Mesenchymal Stem Cells

**DOI:** 10.3390/ijms20020345

**Published:** 2019-01-16

**Authors:** Stefano Martellucci, Costantino Santacroce, Francesca Santilli, Luca Piccoli, Simona Delle Monache, Adriano Angelucci, Roberta Misasi, Maurizio Sorice, Vincenzo Mattei

**Affiliations:** 1 Laboratory of Experimental Medicine and Environmental Pathology, Rieti University Hub “Sabina Universitas”, 02100 Rieti, Italy; s.martellucci@sabinauniversitas.it (S.M.); costantinosantacroce@tiscali.it (C.S.); f.santilli@sabinauniversitas.it (F.S.); 2 Department of Experimental Medicine, “Sapienza” University, 00161 Rome, Italy; roberta.misasi@uniroma1.it (R.M.); maurizio.sorice@uniroma1.it (M.S.); 3 Department of Science Dentistry and Maxillofacial, “Sapienza” University, 00161 Rome, Italy; luca.piccoli@uniroma1.it; 4 Department of Biotechnological and Applied Clinical Sciences, University of L’Aquila, 67100 L’Aquila, Italy; simona.dellemonache@univaq.it (S.D.M.); adriano.angelucci@univaq.it (A.A.)

**Keywords:** cellular prion protein, shed prion protein, recombinant prion protein, prions, lipid rafts, dental pulp-derived stem cells, mesenchymal stem cells, neural stem cells, adult neurogenesis, neuronal differentiation

## Abstract

Human Dental Pulp Stem Cells (hDPSCs) represent a type of adult mesenchymal stem cells that have the ability to differentiate in vitro in several lineages such as odontoblasts, osteoblasts, chondrocytes, adipocytes and neurons. In the current work, we used hDPSCs as the experimental model to study the role of recombinant prion protein 23–231 (recPrP^C^) in the neuronal differentiation process, and in the signal pathway activation of ERK 1/2 and Akt. We demonstrated that recPrP^C^ was able to activate an intracellular signal pathway mediated by extracellular-signal-regulated kinase 1 and 2 (ERK 1/2) and protein kinase B (Akt). Moreover, in order to understand whether endogenous prion protein (PrP^C^) was necessary to mediate the signaling induced by recPrP^C^, we silenced PrP^C^, demonstrating that the presence of endogenous PrP^C^ was essential for ERK 1/2 and Akt phosphorylation. Since endogenous PrP^C^ is a well-known lipid rafts component, we evaluated the role of these structures in the signal pathway induced by recPrP^C^. Our results suggest that lipid rafts integrity play a key role in recPrP^C^ activity. In fact, lipid rafts inhibitors, such as fumonisin B1 and MβCD, significantly prevented ERK 1/2 and Akt phosphorylation induced by recPrP^C^. In addition, we investigated the capacity of recPrP^C^ to induce hDPSCs neuronal differentiation process after long-term stimulation through the evaluation of typical neuronal markers expression such as B3-Tubulin, neurofilament-H (NFH) and growth associated protein 43 (GAP43). Accordingly, when we silenced endogenous PrP^C^, we observed the inhibition of neuronal differentiation induced by recPrP^C^. The combined data suggest that recPrP^C^ plays a key role in the neuronal differentiation process and in the activation of specific intracellular signal pathways in hDPSCs.

## 1. Introduction

The cellular form of prion protein (PrP^C^) is a cell surface glycosylphosphatidyl-inositol (GPI)-anchored glycoprotein that was first identified as a molecule that is able to bind Cu^2+^ in vitro [1]. PrP^C^ is highly conserved in mammalians and it is present in all nucleated cells, although it is mainly expressed in the central and peripheral nervous system [2].

PrP^C^ is located within lipid rafts [3,4], sphingolipid-rich membrane micro domains, and it is present in several kind of cells such as neural and lymphocytic cells [5,6,7]. The membrane-bound isoform can act as a cell surface receptor, or as a co-receptor, recruiting downstream signal transduction pathways [8,9,10,11]. PrP^C^ is involved in a wide range of cellular processes, such as synaptic plasticity, neurite regulation, calcium homeostasis, copper metabolism, apoptosis and cellular resistance to oxidative stress [12,13,14,15,16]. Recent evidence suggests that PrP^C^ plays a possible in the neuronal differentiation processes of stem cells [17,18,19,20,21].

In a previous work, we used human dental pulp stem cell (hDPSCs) as the experimental model to develop an in vitro system used to study proliferation and differentiation process [22]. hDPSCs represent a kind of adult mesenchymal stem cells that show the ability to differentiate in vitro in several multilineages such as odontoblasts, osteoblasts, chondrocytes, adipocytes and neurons [23,24,25,26,27,28,29,30]. The fact that hDPSCs can express basally PrP^C^ [21] and show the ability to differentiate into neuronal-like cells [31] or dopaminergic neuron-like cells [32], prompt it to be considered as a cellular model for the study of neurodegenerative diseases, such as Alzheimer, Parkinson and Huntington diseases [22,33,34].

Several authors showed that PrP^C^ may be subjected to post translational proteolytic processing, including shedding α-β-γ cleavage [35,36,37,38]. These cleavage events have been shown to regulate its physiological functions, produce biologically active fragments such as N1, N2, N3, shed PrP and potentially influence the course of prion diseases [39]. In particular, it has been shown that the shedding of PrP^C^ near the glycosylphosphatidylinositol (GPI) anchor releases almost full-length prion protein from the cell [40] and this phenomenon could be used to regulate the levels of PrP^C^ on the plasma membrane in response to different stimuli [37,39]. Shed PrP^C^ was first identified in the preparation of prion-infected hamster brains where 15% of total PrP^Sc^ was found to end with Gly228 [41]. An identical cleavage site, between Gly228 and Arg229, and the responsible protease were later also found [42] and confirmed [38] for murine PrP^C^ using recombinant human ADAM10 [43]. Indeed, shed PrP^C^ has been found in the supernatant of cultured cells [35,40], as well as in human cerebrospinal fluid [44], suggesting a potential physiological role. This phenomenon is not restricted to the nervous system, since lymphoid cells and platelets have also been shown to shed PrP^C^ [6,37,45]. 

It has been shown that, in cell culture, a small fraction of total PrP^C^ is slowly but constitutively shed into the media and that shed PrP^C^ lacks any parts of the GPI-modification [40]. The cleavage products of PrP^C^ could act as soluble trophic factors in autocrine, paracrine or endocrine ways in different human cell types. Furthermore, as reported by Altmeppen et al. [39], the N1 fragment and shed PrP^C^ could potentially bind β-sheet-rich oligomers, thereby blocking their deleterious effects and directing them to phagocytosis and degradation. In this scenario, increased surface expression and shedding of PrP^C^ might be a mechanism that is able to block out the effects of toxic oligomers. To mimic the effect of shed PrP^C^, we used recPrP^C^ (23–231) as a molecular model; indeed, as reported by several authors, the C-terminal cleavage close to the membrane, releases nearly full length PrP^C^ from the cell surface [35,40,41,43,44,46].

To date, the information regarding the role of shed PrP^C^ on different cell types is still poor. In this context, our goal is to evaluate the possible effect of recPrP^C^ in signal pathways and in differentiation process using hDPSCs as cellular model system. 

Moreover, we will evaluate the possible involvement of endogenous PrP^C^ in recPrP^C^ signal pathways and neuronal differentiation process using siRNA PrP to ablate the expression of prion protein. Since membrane-bound PrP^C^ is a well know raft component, we will also evaluate the possible role of lipid rafts in the signal pathway induced by recPrP^C^ using molecules affecting lipid rafts integrity.

## 2. Results

### 2.1. Role of recPrP^C^ in Signal Pathways of hDPSCs

**Figure 1:**
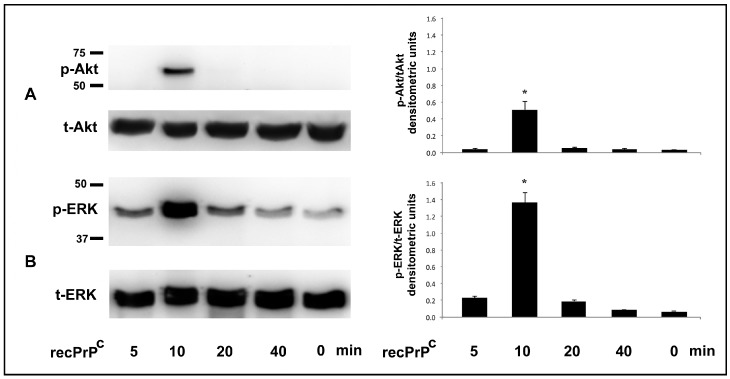
Effect of recPrP^C^ on Akt and ERK Phosphorylation. hDPSCs, untreated or treated with 0.5 µg/mL of recPrP^C^ for 5, 10, 20, 40 min, were analyzed by Western blot using anti-pAkt, anti-total Akt (**A**). anti-pERK1/2 and anti-total ERK1/2 (**B**). Densitometric analysis is shown in the right panel. Results represent the mean ± SD from 3 independent experiments, * *p* recPrP^C^ treated cells <0.01 vs. untreated cells.

In order to evaluate a possible role of human recPrP^C^ in the activation of intracellular signal pathway, hDPSCs were treated with recPrP^C^ for different times: 5, 10, 20, 40 min at 37 °C. Western blot analysis for pERK1-2 and pAkt expression showed the presence of these active forms upon treatment with recPrP^C^ at 10 min (Figure 1A,B). The upregulation of phosphorylated forms, was also confirmed by densitometric analysis (Figure 1, right panels, bar graphs).

### 2.2. Role of Endogenous PrP^C^ in the Modulation of Cell Signaling Induced by recPrP^C^

**Figure 2:**
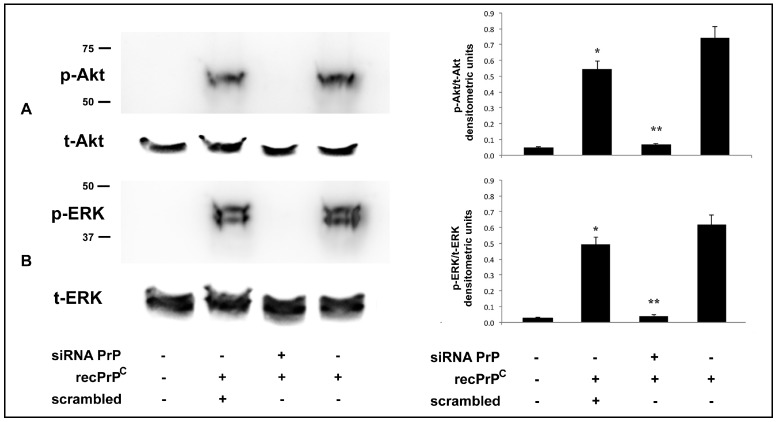
Effect of PrP^C^ silencing on Akt and ERK phosphorylation induced by recPrP^C^. hDPSCs, untreated or treated with 0.5 µg/mL of recPrP^C^ for 10 min in presence or in absence of pre-treatment with siRNA PrP or scrambled siRNA for 72 h, were analyzed by Western blot using anti-pAkt, anti-total Akt (**A**). anti-pERK1/2 and anti-total ERK1/2 (**B**). Densitometric analysis is shown in the right panel. Results represent the mean ± SD from 3 independent experiments, * *p* recPrP^C^ treated cells <0.01 vs. untreated cells, ** *p* siRNA PrP + recPrP^C^ treated cells vs. scrambled + recPrP^C^ treated cells. As control, scrambled siRNA was employed in each experiment.

It is well known that the C-terminal cleavage close to the membrane releases nearly full length PrP^C^ from the cell surface. In order to understand whether the signal pathway induced by recPrP^C^ requires full length endogenous PrP^C^, we used a verified siRNA PrP. With this aim, hDPSCs were pretreated with siRNA PrP for 72 h and, subsequently, were stimulated with recPrP^C^ for 10 min at 37 °C. Western blot analysis demonstrated that pretreatment with siRNA PrP prevented the activation of Akt and ERK 1-2 induced by recPrP^C^ (Figure 2A,B). These results were also confirmed by densitometric analysis (Figure 2, right panels, bar graphs). 

These data indicate that endogenous PrP^C^ is essential for the signal pathway induced by recPrP^C^.

### 2.3. Role of Lipid Rafts in the Modulation of Cell Signaling Induced by recPrP^C^

**Figure 3:**
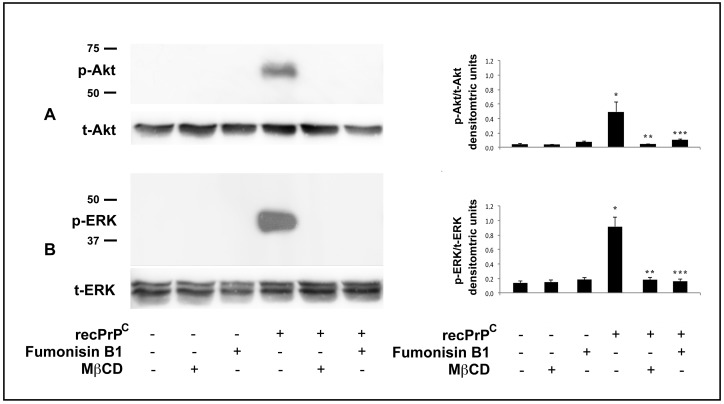
Effect of lipid rafts perturbation on Akt and ERK Phosphorylation induced by recPrP^C^. hDPSCs, untreated or treated with 0.5 µg/mL of recPrP^C^ for 10 min in the presence or in the absence of Fumonisin B1 or MβCD, were analyzed by Western blot using anti-pAkt and anti-total Akt (**A**). anti-pERK1/2 and anti-total ERK1/2 (**B**). Densitometric analysis is shown in the right panel. Results represent the mean ± SD from 3 independent experiments, * *p* recPrP^C^ treated cells <0.01 vs. untreated cells, ** *p* recPrP^C^ treated cells + MβCD <0.01 vs. recPrP^C^ treated cells, *** *p* recPrP^C^ treated cells + Fumonisin B1 <0.01 vs. recPrP^C^ treated cells.

Endogenous PrP^C^ is a well-known raft component, thus we evaluated the role of lipid rafts in the signal pathway induced by recPrP^C^. To analyze the functional role of lipid rafts in recPrP^C^ signal pathways, cells were preincubated with lipid rafts affecting agents, Fumonisin B1 or methyl-β-cyclodextrin (MβCD) and then stimulated with recPrP^C^ for 10 min at 37 °C. Western blot analysis clearly showed that cell pretreatment with either Fumonisin B1 or methyl-β-cyclodextrin, significantly prevents Akt and ERK 1-2 (Figure 3A,B) phosphorylation induced by recPrP^C^, indicating that lipid rafts integrity is essential for recPrP^C^-induced signal pathways of hDPSCs. These results were also confirmed by densitometric analysis (Figure 3, right panels, bar graphs). 

### 2.4. Role of recPrP^C^ in the Neuronal Differentiation of hDPSCs

**Figure 4:**
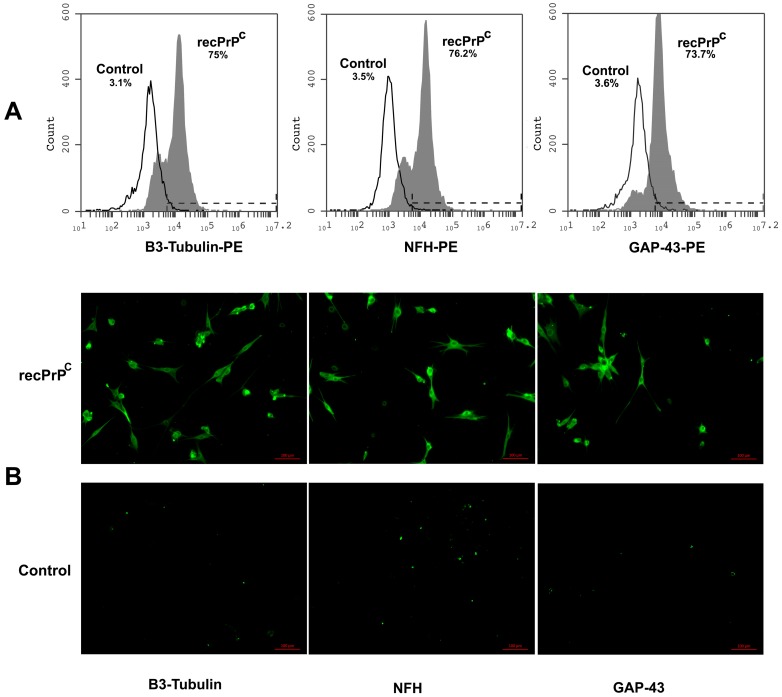
Effect of recPrP^C^ on expression of neuronal markers GAP-43, NFH and B3-Tubulin. hDPSCs, untreated or treated with recPrP^C^ for 14 days, were analyzed by flow cytometry and immunofluorescence analysis using anti-NFH, Anti-B3-Tubulin and anti-GAP-43. (**A**) Flow Cytometry. Histograms represent log fluorescence vs. cell number, gated on cell population of a side scatter/forward scatter (SS/FS) histogram. Cell number is indicated on the y-axis and fluorescence intensity is represented on the x-axis. Each panel was compared with the corresponding secondary antibody as negative control. A representative experiment among 3 is shown. (**B**) Immunofluorescence analysis. Alternatively, hDPSCs were analyzed by immunofluorescence analysis using anti-B3-Tubulin, anti-NFH and anti-GAP-43 and observed with a Zeiss Axio Vert. A1 fluorescence microscope (Zeiss, Oberkochen, Germany). Scale bars, 100 µm.

We further analyzed the possible role of recPrP^C^ in the neuronal differentiation of hDPSCs. With this aim, we performed flow cytometry and immunofluorescence analysis of hDPSCs with recPrP^C^ to verify the expression of typical neuronal markers such as B3-Tubulin, NFH and GAP 43. Flow cytometry analysis showed positive values for all the neuronal antigens under testing (>73%) (Figure 4A). Immunofluorescence analysis confirmed the expression of these typical neuronal markers in hDPSCs stimulated with recPrP^C^ for 14 days, but not in control cells (Figure 4B). 

These findings demonstrate that recPrP^C^ is able to induce neuronal differentiation of hDPSCs. 

### 2.5. Role of Endogenous PrP^C^ in the Neuronal Differentiation Process Induced by recPrP^C^

To analyze whether the effect of recPrP^C^ on neuronal differentiation of hDPSCs requires full length endogenous PrP^C^, hDPSCs were stimulated with recPrP^C^ in the presence or in the absence of siRNA PrP pre-treatment.

**Figure 5:**
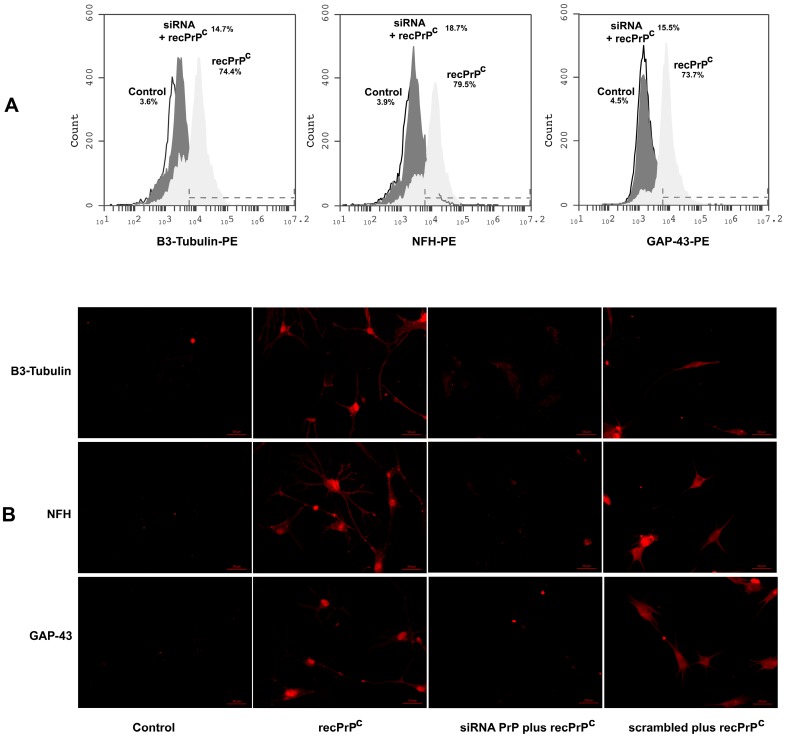
Effect of PrP^C^ silencing on expression of neuronal markers B3-Tubulin, NFH and GAP-43. hDPSCs, stimulated with 0.5 µg/mL of recPrP^C^ for 14 days in the presence or in the absence of pre-treatment with siRNA PrP or scrambled siRNA for 72 h, were analyzed by flow cytometry or immunofluorescence analysis using anti-B3-Tubulin, anti-NFH and anti-GAP-43. (**A**) Flow Cytometry. Histograms represent log fluorescence vs. cell number, gated on cell population of a side scatter/forward scatter (SS/FS) histogram. Cell number is indicated on the y-axis, and fluorescence intensity is represented on the x-axis. Each panel was compared with the corresponding secondary antibody as a negative control. A representative experiment among 3 is shown. (**B**) Immunofluorescence analysis. Alternatively, hDPSCs were analyzed by immunofluorescence analysis using anti-B3-Tubulin, anti-NFH and anti-GAP-43 and, then, observed with a Zeiss Axio Vert. A1 fluorescence microscope. Scale bars, 50 µm.

Our results revealed that the expression of the neuronal markers B3-Tubulin, NFH and GAP 43 of hDPSCs induced by recPrP^C^ was significantly inhibited (*p* < 0.001) by previous silencing of PrP, as revealed by flow cytometry (Figure 5A). These findings were also confirmed by immunofluorescence analysis under the same conditions (Figure 5B). 

This data indicates that the endogenous PrP^C^ is essential for neuronal differentiation induced by recPrP^C^. 

## 3. Discussion

In this study we have investigated the cellular and molecular mechanisms mediated by recPrP^C^ involved in the neuronal differentiation process of mesenchymal stem cells. Our results confirm and extend previous data which suggested that PrP^C^ is involved in the neuronal differentiation process of adult stem cells [21].

Shed PrP^C^ and the cleavage products N1, N2, N3 are biologically active fragments that may potentially influence the course of prion diseases, or participate with other biological processes. Indeed, proteolytic cleavage events may alter either biological functions of PrP^C^ or produce protein fragments harboring specific intrinsic properties, thus contributing to a higher biological complexity [30]. It is well-known that the cleavage of certain key proteins is a highly relevant mechanism with regard to neurodegenerative diseases [43]. In particular, shed PrP^C^ has been found in supernatants of cultured cells [35,40], as well as in human cerebrospinal fluid [44], which is indicative of its physiological role. There are many observations which highlight that this process is not restricted to the nervous system, since lymphoid cells and platelets have also been shown to shed PrP^C^ [6,37,45]. Moreover, in cell culture, a small fraction of total PrP^C^ is slowly but constitutively shed into the media, and shed PrP^C^ lacks any parts of the GPI-modification [40]. Within this context, it may be interesting to understand the role of shed PrP^C^ and its fragments in physiological or pathological conditions.

Our goal was to understand whether a molecule able to mimic shed PrP^C^, i.e., recPrP^C^ (23–231 a.a.), was able to activate a signal pathway in our stem cells experimental model, and whether it may influence neuronal differentiation process. hDPSCs represent a type of adult mesenchymal stem cells that have the ability to differentiate in vitro in several multilineages such as odontoblasts, osteoblasts, chondrocytes, adipocytes and neurons [23,24,25,26,27,28,29,30].

Our results demonstrated that recPrP^C^ was able to activate a signal transduction pathway by phosphorylation of ERK 1/2 and Akt-kinase. A particularly interesting matter, is that this activity required an endogenous PrP^C^ to mediate the way of the signal triggered by recPrP^C.^ In fact, when the same experiment of stimulation with recPrP was conducted in cells with a transient silencing of PrP, the phosphorylation of ERK molecules 1/2 and Akt was significantly reduced.

Previous studies [2,47] reported that PrP^C^ was able to form dimeric structures, both in its physiologic and pathologic status. As previously mentioned, shed PrP^C^ and cleavage products, such as PrP-N1, are stimulated as a consequence of intracellular dimerization. Dimerization of membrane-bound PrP^C^ leads to clustering in multimolecular complexes and serves to regulate different aspects of neuronal homeostasis, whereas intracellular dimerization appears to be the most relevant event in neuroprotection, via N1 and C1 prion metabolites [2]. Indeed, the dimerization stimulates α-cleavage and thus the production of the neuroprotective fragments. 

Since membrane-bound PrP^C^ is a well know raft component, we also evaluated the possible role of lipid rafts in the signal pathway induced by recPrP^C^. We used molecules affecting lipid rafts microdomains integrity, such as Fumonisin B1 or MβCD, revealing that cell pretreatment with these molecules significantly prevented ERK 1/2 and Akt phosphorylation induced by recPrP^C^. These results suggest that lipid rafts integrity is essential for recPrP^C^-induced signal pathways in hDPSCs, and that both gangliosides and cholesterol play a key role. These data are in agreement with our previous study [48], where we demonstrated that lipid rafts integrity was essential for neuronal differentiation process of hDPSCs induced by epidermal growth factor and basic fibroblast growth factor (EGF/bFGF). 

In conclusion, our findings suggest that recPrP^C^ play a key role in the neuronal differentiation process and in the activation of specific intracellular signal pathways. Cellular prion protein and its cleavage products represent enigmatic molecules, since their polymorphic behavior is still not fully understood. We suggest that membrane-bound PrP^C^ and its cleavage products play a role in a type of dynamic equilibrium dependent on cellular conditions, and is able to function as sensors influencing signal pathways through autocrine, paracrine or endocrine mechanisms.

Better knowledge on cellular and molecular pathways playing a role in differentiation mechanisms mediated by endogenous and/or recombinant prion protein could allow to clarify the involvement of stem cells in nerve regeneration processes in order to better direct their use in regenerative medicine.

## 4. Materials and Methods

### 4.1. Research Ethics

To obtain hDPSCs, third molars included were excised from patients aged 13–19 years [48,49]. All subjects gave their informed consent for inclusion before their participation in the study, which was conducted in accordance with the Declaration of Helsinki, and the protocol was approved on 26 January 2017 by the Ethics Committee of “Sapienza” University (Project identification code CE:4336).

### 4.2. Isolation of Stem Cells Derived from Human Dental Pulp

hDPSCs were isolated and cultured as described in previous works [21,48,49] and, subsequently, they were maintained in Dulbecco’s Modified Eagle’s Medium low glucose (DMEM-L), containing 100 units/mL penicillin, 10 mg/mL streptomycin, plus 0.1% amphotericin (Sigma-Aldrich, Milan, Italy), plus fetal bovine serum (FBS) qualified 10% (Life Technologies, Monza, Italy), at 37 °C in humified CO_2_ atmosphere.

### 4.3. Treatments

Before treatments, hDPSCs were cultured up to 28 days from the pulp separation. For cell signaling analysis, hDPSCs were stimulated with recPrP^C^ (0.5 μg/mL) (Jena Bioscience, Jena, Germany) several times (5, 10, 20, 40 min) at 37 °C in 5% CO_2_. To understand the implication of lipid rafts in the signaling of recPrP^C^, hDPSCs were pretreated with 30 μM Fumonisin B1 (Sigma-Aldrich, Milan, Italy), a compound that blocks the synthesis of sphingolipids, for 24 h at 37 °C or, alternatively, with 5 mM methyl-β-cyclodextrin (MβCD) (Sigma-Aldrich, Milan, Italy), a compound which is known to induce cholesterol efflux from the membrane, for 30 min at 37 °C. Moreover, to assess the role of endogenous PrP^C^ in recPrP^C^ signal pathways and neuronal differentiation induction, hDPSCs were pretreated with siRNA PrP for 72 h as described extensively below. To evaluate the role of recPrP^C^ in neuronal differentiation process, hDPSCs were stimulated with recPrP^C^ (0.5 μg/mL) for a long exposure time (14 days) changing the media every 4 days.

### 4.4. Knockdown PrP^C^ by siRNA

hDPSCs were seeded (5 × 10^4^ cells/mL) 6-well plates, in DMEM-L containing serum and antibiotics. Twenty-four hours after seeding, cells were transfected with 5 nM siRNA PrP (Flexitube GeneSolution GS5621 for PRNP), using HiPerFect Transfection Reagent (Qiagen, Valencia, CA, USA), according to the manufacturer’s instructions. As experimental control, cells were also transfected with 5 nM scrambled siRNA (AllStars Negative Control—Qiagen). We verified by TLC analysis that PrP siRNA did not affect both cholesterol and ganglioside content, thus not affecting lipid rafts (data not shown). After 72 h, cells were incubated with recPrP^C^ for 10 min (to evaluate signal pathways) or 14 days (to evaluate neuronal differentiation process) at 37 °C. For long time exposure, (14 days) every 4 days the siRNA PrP was replaced. PrP^C^ expression was verified by flow cytometry analysis using mouse anti-PrP SAF32 mAb (Spi-Bio, Bertin Pharma, France) (Figure S1).

### 4.5. Western Blot Analysis

hDPSCs were lysed in lysis buffer containing 0.1% Triton X-100, 10 mM Tris-HCl (pH 7.5), 150 mM NaCl, 5 mM EDTA, 1 mM Na_3_VO_4_ and 75 U of aprotinin and allowed to stand for 20 min at 4 °C. The cell suspension was mechanically disrupted by Dounce homogenization (10 strokes). The lysate was centrifuged for 5 min at 1300× *g* to eliminate nuclei and large cellular debris. After protein concentration analysis by Bradford Dye Reagent assay (Bio-Rad, Milano, Italia), the lysate was tested with sodium dodecyl sulfate-polyacrylamide gel electrophoresis (SDS-PAGE). Subsequently, the proteins were electrophoretically transferred to PVDF membranes (Bio-Rad, Milan, Italia) which were blocked with 5% nonfat dried milk (Bio-Rad, Milan, Italia), or Bovine Serum Albumine (BSA) (Sigma-Aldrich, Milan, Italy), in TBS (Bio-Rad, Milan, Italia), containing 0.05% Tween 20 (Bio-Rad, Milan, Italia), and probed with rabbit anti-p-ERK1/2 pAb, rabbit anti-total ERK1/2 pAb, rabbit anti-p-Akt pAb, rabbit anti-total Akt pAb. Antibodies were visualized with horseradish peroxidase (HRP)-conjugated anti-rabbit IgG (Cell Signaling Technology Danvers, MA, USA) and immunoreactivity assessed by chemiluminescence reaction, using the ECL detection system (ThermoFisher Scientific, Rockford, IL, USA). Densitometric scanning analysis was accomplished with NIH Image 1.62 software by Mac OS X (Apple Computer International).

### 4.6. Flow Cytometry Analysis

Flow cytometry was used to quantify neuronal antigen expression on hDPSCs after a long period of stimulation with recPrP^C^. Briefly, hDPSCs untreated or treated with siRNA PrP or scrambled siRNA for 72 h, were stimulated with recPrP^C^ for 14 days as described below and fixed with 4% paraformaldehyde and permeabilized by 0.1% (*v/v*) Triton X-100. After washing, cells were incubated with mouse anti-B3-Tubulin mAb, mouse anti-NFH mAb (Cell Signaling Technology Danvers, MA, USA) and mouse anti-GAP43 mAb (Sigma-Aldrich, Milan, Italy) for 1 h at 4 °C, followed by PE-conjugated anti-mouse IgG H&L (Abcam, Cambridge, MA, USA) for additional 30 min. All samples were analyzed with a FACScan cytometer (BD Accuri C6 Flow cytometer) equipped with a blue laser (488 nm) and a red laser (640 nm). At least 20,000 events were acquired.

### 4.7. Immunofluorescence Analysis

hDPSCs were seeded (2 × 10^4^ cells/mL) 6-well plates, in DMEM-L containing serum and antibiotics. Twenty-four hours after seeding, hDPSCs untreated or treated with siRNA PrP or scrambled for 72 h were stimulated with recPrP^C^ for 14 days and tested for immunofluorescence analysis. Briefly, hDPSCs untreated or treated as above were fixed with 4% paraformaldehyde and permeabilized by 0.1% (*v/v*) Triton X-100. After washing, cells were incubated with mouse anti-B3-Tubulin mAb, mouse anti-NFH mAb (Cell Signaling Technology Danvers, MA, USA) and mouse anti-GAP43 mAb (Sigma-Aldrich, Milan, Italy) for 1 h at 4 °C, followed by anti-mouse alexa fluor 488 or anti-mouse alexa fluor 594 (Cell Signaling Technology Danvers, MA, USA) for additional 30 min. Finally, cells were observed with a Zeiss Axio Vert. A1 fluorescence microscope (Zeiss, Oberkochen, Germany).

### 4.8. Statistical Analysis 

Western blot images were subjected to densitometric scanning analysis, performed by Mac OS X (Apple Computer International), using NIH Image 1.62 software. The data were analyzed using one-way analysis of variance (ANOVA) after Bartlett’s test for the homogeneity of variances and Kolmogorov-Smirnov’s test for the Gaussian distribution and followed by Newman-Keuls multiple-comparison test or, when appropriate, with Student’s t-test (StatView for Macintosh; SAS Institute, Cary, NC, USA). All data were verified in at least 3 different experiments and reported as mean ± standard deviation (SD). Only *p* values of <0.01 were considered as statistically significant. 

## Supplementary Materials

Supplementary materials can be found at http://www.mdpi.com/1422-0067/20/2/345/s1.

## References

[1] Brown D.R., Qin K., Herms J.W., Madlung A., Manson J., Strome R., Fraser P.E., Kruck T., von Bohlen A., Schulz-Schaeffer W. (1997). The cellular prion protein binds copper in vivo. Nature.

[2] Mattei V., Martellucci S., Santilli F., Manganelli V., Garofalo T., Candelise N., Caruso A., Sorice M., Scaccianoce S., Misasi R. (2017). Morphine Withdrawal Modifies Prion Protein Expression in Rat Hippocampus. PLoS ONE.

[3] Lewis V., Hooper N.M. (2011). The role of lipid rafts in prion protein biology. Front Biosci..

[4] Sorice M., Mattei V., Tasciotti V., Manganelli V., Garofalo T., Misasi R. (2012). Trafficking of PrP^C^ to mitochondrial raft-like microdomains during cell apoptosis. Prion.

[5] Prusiner S.B., Scott M.R., DeArmond S.J., Cohen F.E. (1998). Prion protein biology. Cell.

[6] Parizek P., Roeckl C., Weber J., Flechsig E., Aguzzi A., Raeber A.J. (2001). Similar turnover and shedding of the cellular prion protein in primary lymphoid and neuronal cells. J. Biol. Chem..

[7] Mattei V., Garofalo T., Misasi R., Circella A., Manganelli V., Lucania G., Pavan A., Sorice M. (2004). Prion protein is a component of the multimolecular signaling complex involved in T cell activation. FEBS Lett..

[8] Mouillet-Richard S., Ermonval M., Chebassier C., Laplanche J.L., Lehmann S., Launay J.M., Kellermann O. (2000). Signal transduction through prion protein. Science.

[9] Toni M., Spisni E., Griffoni C., Santi S., Riccio M., Lenaz P., Tomasi V. (2006). Cellular prion protein and caveolin-1 interaction in a neuronal cell line precedes Fyn/Erk 1/2 signal transduction. J. Biomed. Biotechnol..

[10] Llorens F., Carulla P., Villa A., Torres J.M., Fortes P., Ferrer I., del Río J.A. (2013). PrP(C) regulates epidermal growth factor receptor function and cell shape dynamics in Neuro2a cells. J. Neurochem..

[11] Hirsch T.Z., Martin-Lannerée S., Mouillet-Richard S. (2017). Functions of the Prion Protein. Prog. Mol. Biol. Transl. Sci..

[12] Hu W., Kieseier B., Frohman E., Eagar T.N., Rosenberg R.N., Hartung H.P., Stüve O. (2008). Prion proteins: Physiological functions and role in neurological disorders. J. Neurol. Sci..

[13] Mattei V., Matarrese P., Garofalo T., Tinari A., Gambardella L., Ciarlo L., Manganelli V., Tasciotti V., Misasi R., Malorni W. (2011). Recruitment of cellular prion protein to mitochondrial raft-like microdomains contributes to apoptosis execution. Mol. Biol. Cell.

[14] Garofalo T., Manganelli V., Grasso M., Mattei V., Ferri A., Misasi R., Sorice M. (2015). Role of mitochondrial raft-like microdomains in the regulation of cell apoptosis. Apoptosis.

[15] Wulf M.A., Senatore A., Aguzzi A. (2017). The biological function of the cellular prion protein: An update. BMC Biol..

[16] Linden R. (2017). The Biological Function of the Prion Protein: A Cell Surface Scaffold of Signaling Modules. Front Mol. Neurosci..

[17] Kanaani J., Prusiner S.B., Diacovo J., Baekkeskov S., Legname G. (2005). Recombinant prion protein induces rapid polarization and development of synapses in embryonic rat hippocampal neurons in vitro. J. Neurochem..

[18] Steele A.D., Emsley J.G., Ozdinler P.H., Lindquist S., Macklis J.D. (2006). Prion protein (PrPc) positively regulates neural precursor proliferation during developmental and adult mammalian neurogenesis. Proc. Natl. Acad. Sci. USA.

[19] Miranda A., Ramos-Ibeas P., Pericuesta E., Ramirez M.A., Gutierrez-Adan A. (2013). The role of prion protein in stem cell regulation. Reproduction.

[20] Lee Y.J., Baskokov I.V. (2014). The cellular form of the prion protein guides the differentiation of human embryonic stem cell into neuron-, oligodendrocyte- and astrocyte-committed lineages. Prion.

[21] Martellucci S., Manganelli V., Santacroce C., Santilli F., Piccoli L., Sorice M., Mattei V. (2018). Role of Prion protein-EGFR multimolecular complex during neuronal differentiation of human dental pulp-derived stem cells. Prion.

[22] Mediano D.R., Sanz-Rubio D., Ranera R., Bolea I., Martin-Burriel I. (2015). The potential of mesenchymal stem cell in prion research. Zoonoses Public Health.

[23] Gronthos S., Brahim J., Li W., Fisher L.W., Cherman N., Boyde A., DenBesten P., Robey P.G., Shi S. (2002). Stem cell properties of human dental pulp stem cells. J. Dent. Res..

[24] Arthur A., Rychkov G., Shi S., Koblar S.A., Gronthos S. (2008). Adult human dental pulp stem cells differentiate toward functionally active neurons under appropriate environmental cues. Stem Cells.

[25] Suchanek J., Soukup T., Visek B., Ivancakova R., Kucerova L., Mokry J. (2009). Dental pulp stem cells and their characterization. Biomed. Pap. Med. Fac. Univ. Palacky Olomouc. Czech Repub..

[26] Koyama N., Okubo Y., Nakao K., Bessho K. (2009). Evaluation of pluripotency in human dental pulp cells. J. Oral Maxillofac Surg..

[27] Lee S.H., Ryu J.S., Lee J.W., Kwak D.H., Ko K., Choo Y.K. (2010). Comparison of ganglioside expression between human adipose- and dental pulp-derived stem cell differentiation into osteoblasts. Arch. Pharm. Res..

[28] Bieberich E. (2012). It’s a lipid’s world: Bioactive lipid metabolism and signaling in neural stem cell differentiation. Neurochem. Res..

[29] Atari M., Gil-Recio C., Fabregat M., García-Fernández D., Barajas M., Carrasco M.A., Jung H.S., Alfaro F.H., Casals N., Prosper F. (2012). Dental pulp of the third molar: A new source of pluripotent-like stem cells. J. Cell Sci..

[30] Young F.I., Telezhkin V., Youde S.J., Langley M.S., Stack M., Kemp P.J., Waddington R.J., Sloan A.J., Song B. (2016). Clonal heterogeneity in the neuronal and glial differentiation of dental pulp stem/progenitor cells. Stem Cells Int..

[31] Ullah I., Subbarao R.B., Kim E.J., Bharti D., Jang S.J., Park J.S., Shivakumar S.B., Lee S.L., Kang D., Byun J.H. (2016). In vitro comparative analysis of human dental stem cells from a single donor and its neurodifferentiation potential evaluated by electrophysiology. Life Sci..

[32] Chun S.Y., Soker S., Jang Y.J., Kwon T.G., Yoo E.S. (2016). Differentiation of human dental pulp stem cells into dopaminergic neuron-like cells in vitro. J. Korean Med. Sci..

[33] Park S., Kim E., Koh S.E., Maeng S., Lee W.D., Lim J., Shim I., Lee Y.J. (2012). Dopaminergic differentiation of neural progenitors derived from placental mesenchymal stem cells in the brains of Parkinson’s disease model rats and alleviation of asymmetric rotational behaviour. Brain Res..

[34] Nesti C., Pardini S., Barachini S., D’Alessandro D., Siciliano G., Murri L., Petrini M., Vaglini F. (2011). Human dental pulp stem cells protect mouse dopaminergic neurons against MPP+ orrotenone. Brain Res..

[35] Harris D.A., Huber M.T., van Dijken P., Shyng S.L., Chait B.T., Wang R. (1993). Processing of a cellular prion protein: Identification of N- and C-terminal cleavage sites. Biochemistry.

[36] Watt N.T., Taylor D.R., Gillott A., Thomas D.A., Perera W.S., Hooper N.M. (2005). Reactive oxygen species-mediated beta-cleavage of the prion protein in the cellular response to oxidative stress. J. Biol. Chem..

[37] Altmeppen H.C., Prox J., Puig B., Dohler F., Falker C., Krasemann S., Glatzel M. (2013). Roles of endoproteolytic α-cleavage and shedding of the prion protein in neurodegeneration. FEBS J..

[38] McDonald A.J., Dibble J.P., Evans E.G., Millhauser G.L. (2014). A new paradigm for enzymatic control of α-cleavage and β-cleavage of the prion protein. J. Biol. Chem..

[39] Altmeppen H.C., Puig B., Dohler F., Thurm D.K., Falker C., Krasemann S., Glatzel M. (2012). Proteolytic processing of the prion protein in health and disease. Am. J. Neurodegener Dis..

[40] Borchelt D.R., Rogers M., Stahl N., Telling G., Prusiner S.B. (1993). Release of the cellular prion protein from cultured cells after loss of its glycoinositol phospholipid anchor. Glycobiology.

[41] Stahl N., Borchelt D.R., Prusiner S.B. (1990). Differential release of cellular and scrapie prion proteins from cellular membranes by phosphatidylinositol-specific phospholipase C. Biochemistry.

[42] Taylor D.R., Parkin E.T., Cocklin S.L., Ault J.R., Ashcroft A.E., Turner A.J., Hooper N.M. (2009). Role of ADAMs in the ectodomain shedding and conformational conversion of the prion protein. J. Biol. Chem..

[43] Linsenmeier L., Altmeppen H.C., Wetzel S., Mohammadi B., Saftig P., Glatzel M. (2017). Diverse functions of the prion protein—Does proteolytic processing hold the key?. Biochim. Biophys. Acta Mol. Cell Res..

[44] Tagliavini F., Prelli F., Porro M., Salmona M., Bugiani O., Frangione B. (1992). A soluble form of prion protein in human cerebrospinal fluid: Implications for prion-related encephalopathies. Biochem. Biophys. Res. Commun..

[45] Perini F., Vidal R., Ghetti B., Tagliavini F., Frangione B., Prelli F. (1996). PrP27-30 is a normal soluble prion protein fragment released by human platelets. Biochem. Biophys. Res. Commun..

[46] Zhang C.C., Steele A.D., Lindquist S., Lodish H.F. (2006). Prion protein is expressed on long-term repopulating hematopoietic stem cells and is important for their self-renewal. Proc. Natl. Acad. Sci. USA.

[47] Rigter A., Langeveld J.P.M., Zijderveld F.G.V., Bossers A. (2010). Prion protein self-interactions: A gateway to novel therapeutic strategies?. Vaccine.

[48] Mattei V., Santacroce C., Tasciotti V., Martellucci S., Santilli F., Manganelli V., Piccoli L., Misasi R., Sorice M., Garofalo T. (2015). Role of lipid rafts in neuronal differentiation of dental pulp-derived stem cells. Exp. Cell Res..

[49] Martellucci S., Santacroce C., Manganelli V., Santilli F., Piccoli L., Cassetta M., Misasi R., Sorice M., Mattei V. Isolation, Propagation and Prion Protein Expression During Neuronal Differentiation Process of Human Dental Pulp Stem Cells. J. Vis. Exp..

