# Supplementary material for: Cellular and Molecular Mechanisms Mediated by recPrPC Involved in the Neuronal Differentiation Process of Mesenchymal Stem Cells
Source: Int J Mol Sci. 2019 Jan 16;20(2):345. doi: 10.3390/ijms20020345 (PMC6358746; doi:10.3390/ijms20020345)
Supplement: Supplementary file 1 [file ijms-20-00345-s001.pdf]

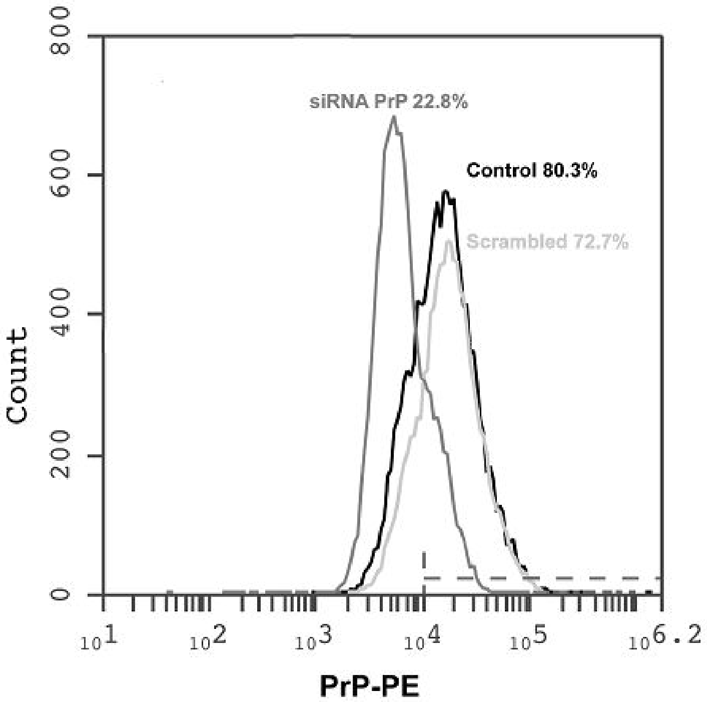

Figure S1. Endogenous PrP^C expression in siRNA PrP and Scrambled-treated hDPSCS. hDPSCs, untreated or treated with siRNA PrP or Scrambled siRNA for 72 h, were analyzed by Flow Cytometry analysis using mouse anti-PrP SAF32 mAb. Flow Histograms represent log fluorescence *vs* cell number, gated on cell population of a side scatter/forward scatter (SS/FS) histogram. Cell number is indicated on the y-axis and fluorescence intensity is represented on the x-axis. The panel was compared with the corresponding secondary antibody as negative control. A representative experiment among 3 is shown.
